# Devil Declines and Catastrophic Cascades: Is Mesopredator Release of Feral Cats Inhibiting Recovery of the Eastern Quoll?

**DOI:** 10.1371/journal.pone.0119303

**Published:** 2015-03-11

**Authors:** Bronwyn A. Fancourt, Clare E. Hawkins, Elissa Z. Cameron, Menna E. Jones, Stewart C. Nicol

**Affiliations:** School of Biological Sciences, University of Tasmania, Hobart, Tasmania, Australia; University of Queensland, AUSTRALIA

## Abstract

The eastern quoll (*Dasyurus viverrinus*) is a medium-sized Australian marsupial carnivore that has recently undergone a rapid and severe population decline over the 10 years to 2009, with no sign of recovery. This decline has been linked to a period of unfavourable weather, but subsequent improved weather conditions have not been matched by quoll recovery. A recent study suggested another mechanism: that declines in Tasmanian devil (*Sarcophilus harrisii*) populations, due to the spread of the fatal Devil Facial Tumour Disease, have released feral cats (*Felis catus*) from competitive suppression, with eastern quoll declines linked to a subsequent increase in cat sightings. Yet current evidence of intraguild suppression among devils, cats and quolls is scant and equivocal. We therefore assessed the influences of top-down effects on abundance and activity patterns among devils, feral cats and eastern quolls. Between 2011 and 2013, we monitored four carnivore populations using longitudinal trapping and camera surveys, and performed camera surveys at 12 additional sites throughout the eastern quoll’s range. We did not find evidence of a negative relationship between devil and cat abundance, nor of higher cat abundance in areas where devil populations had declined the longest. Cats did not appear to avoid devils spatially; however, there was evidence of temporal separation of cat and devil activity, with reduced separation and increasing nocturnal activity observed in areas where devils had declined the longest. Cats and quolls used the same areas, and there was no evidence that cat and quoll abundances were negatively related. Temporal overlap in observed cat and quoll activity was higher in summer than in winter, but this seasonal difference was unrelated to devil declines. We suggest that cats did not cause the recent quoll decline, but that predation of juvenile quolls by cats could be inhibiting low density quoll populations from recovering their former abundance through a ‘predator pit’ effect following weather-induced decline. Predation intensity could increase further should cats become increasingly nocturnal in response to devil declines.

## Introduction

Top predators can function as keystone species, influencing ecosystem composition and functioning through top-down processes [1, 2]. Both top predators and other large predators can limit the abundance, distribution and behaviour of sympatric medium-sized predators, or ‘mesopredators’, which in turn could influence smaller predators, prey and plant communities [1, 3, 4]. Top predators can suppress the abundance of mesopredators through direct killing [5]. They can also suppress mesopredator activity by causing them to shift their spatial or temporal activity to partition limited resources or avoid aggressive interactions with larger predators [3, 5–7]. Such shifts could lead to fitness reductions [8] which could in turn translate to decreased mesopredator abundance [6]. Conversely, declining abundance of a top predator can release mesopredators from competitive pressures, allowing them to increase in abundance or adopt spatial and temporal shifts in activity that could increase their impact on competitors and prey species [9–11]. The direction, magnitude, rapidity and duration of responses, however, are context dependant and therefore differ markedly between systems [9, 12–15].

In Australia’s island state of Tasmania (68 400 km^2^), the Tasmanian devil (*Sarcophilus harrisii*; 7–11 kg) has been hypothesised to suppress smaller mesopredators such as the feral cat (*Felis catus*; 2–6 kg) [16], with similar size-based suppression observed in predator communities around the world [11, 17]. The devil is the island’s largest mammalian predator, following the extinction of the island’s apex predator, the thylacine (*Thylacinus cynocephalus*), almost 80 years ago [18, 19]. However, the species’ differing feeding ecologies [20–22] suggests that their ecological function would also differ.

It has been suggested that the functional loss of devils from Tasmanian ecosystems could release feral cats, allowing them to increase in abundance or extend their activity to intensify predation on other species, including smaller predators such as the eastern quoll (*Dasyurus viverrinus*) [16]. Since 1996, devil populations have undergone rapid and severe decline due to the spread of Devil Facial Tumour Disease (DFTD) [23]. The largest absolute changes in devil abundance would be expected to occur in the first few years following disease arrival [24]. Adults have been observed to decline by around 50% per year [25], and population densities reduced by 90% or more within 10 years of DFTD emergence at many sites [24]. These changes could vary across the landscape, due at least in part to variant forms of the disease [26]. At some sites (such as Cradoc and Judbury surveyed in the current study), no cases of DFTD have been recorded, despite the disease having been recorded in the region up to eight years earlier. Transmission of DFTD is strongly frequency-dependent [24]: even at low densities, populations have shown the same prevalence of the disease, and therefore proportionate rate of decline. However, more recent findings indicate that at extremely low densities, prevalence (and therefore rate of decline) could be reduced (Sam Fox, Save the Tasmanian Devil Program (STTDP), pers. comm.).

Evidence for a change in abundance of feral cats following devil decline is currently scant and unclear, although there are some indications that devils might be influencing cat activity. Hollings, et al. [27] showed an increase in feral cat sightings from spotlight surveys in NE Tasmania, coinciding with the arrival of DFTD in the region and subsequent declines in devil abundance. Suggesting that this increase in sightings reflected an increase in cat abundance, they acknowledged that behavioural shifts could also explain some of the observed increase due to changes in detectability, although it was not possible to distinguish between the two from their data set [27]. Contrary to their findings in NE Tasmania, the authors also observed a decrease in cat sightings following DFTD arrival in central northern Tasmania that was positively associated with sightings of native medium-sized mammals and invasive rabbits (potential prey species), indicating that responses of cats were not consistent across regions and that bottom-up processes such as food availability might also be important in driving cat populations. More robust camera studies (i.e. of longer continuous duration, less sensitive to behavioural effects on detectability, and accounting for imperfect detection) have all found non-negative relationships between devils and cats. Saunders [28] found a strong positive association between cat occupancy and devil abundance in DFTD-free areas supporting high devil abundance in NW Tasmania, while Troy [29] found no numerical or behavioural relationship between devils and cats across NE, NW and southern Tasmania. Similarly, Lazenby [30] found that feral cat population trends did not appear to be negatively affected by devils in Southern Tasmania. However, Lazenby and Dickman [31] found that cats were detected less frequently on cameras where devils were detected, suggesting that cats might avoid areas with higher devil activity, although devils were detected more often at cameras where cats were detected. Spatial or temporal separation would minimise the likelihood of agonistic encounters [5] and thus indicates a reduced risk of interference competition for cats, thereby enabling them to coexist with devils. Similar separation has been observed among a number of sympatric carnivores globally [3, 32–34].

A decline in eastern quoll abundance has been linked with increasing cat abundance or activity, inferred from an increase in feral cat sightings from spotlight surveys [27]. The eastern quoll is a medium-sized (0.85–2.00 kg) marsupial carnivore that has recently undergone severe and rapid decline across Tasmania [35]. The species is extinct on the Australian mainland and survives only in Tasmania [36] where it has, until recently, been considered abundant and secure [37]. In the 10 years to 2009, the species has declined by more than 50% with no sign of recovery [35]. Cats and eastern quolls have coexisted in Tasmania for over 200 years [38] without obvious detrimental impacts of cats on quolls; however it was suggested that, prior to the devil decline, the eastern quoll had been indirectly protected from these impacts by devils, through their suppression of feral cats [27]. A recent study found the prevalence of *Toxoplasma gondii* (a cat-borne parasite) was significantly higher in declined quoll populations than in a stable quoll population [39]. While *T*. *gondii* infection did not affect quoll survival, higher prevalence at sites where quolls had declined signalled higher feral cat activity, implying an increased risk of cat predation and/or competition at those sites [39]. However the interactions between cats and eastern quolls have not been investigated.

Another recent study modelled the effects of climatic fluctuations on the eastern quoll’s distribution and abundance, and suggested that a period of unsuitable weather (high precipitation and warm winter temperatures) had caused a rapid decline in quoll abundance between 2001 and 2003 (B. Fancourt pers. comm.). However, while favourable weather conditions have since returned, quoll abundance has not recovered, suggesting that some other factor unrelated to weather is preventing recovery. The hypothesised increase in cat abundance or activity following devil decline could explain the inability of quolls to recover.

We therefore investigated the influences of top-down effects on abundance and activity patterns among devils, feral cats and eastern quolls across the quoll’s range, at sites where DFTD had first been reported in the region between 5 and 16 years earlier. We used a combination of trapping and remote camera surveys to investigate whether devils suppress cat abundance or activity, and whether cats suppress eastern quoll abundance or activity. We made four predictions: (1) feral cat abundance would be negatively related to devil abundance, and would be highest in areas where devil populations had declined the longest; (2) feral cat activity would be separated temporally and/or spatially from devil activity, and this separation would be less in areas with reduced devil activity; (3) eastern quoll abundance would be negatively related to cat abundance, and quoll abundance would be lower in areas where devil populations had declined the longest; and (4) feral cat activity would closely match eastern quoll activity in areas with reduced quoll abundance, but would differ in areas with high quoll abundance. We discuss the importance of our findings in terms of potential mesopredator release in the functional absence of a top predator, the Tasmanian devil, and the possible contribution of feral cats to the eastern quoll decline or inhibiting their recovery.

## Materials and Methods

### Ethics statement

This study was carried out in accordance with the University of Tasmania Animal Ethics Committee Permit #A11655 with permission from the Tasmanian Department of Primary Industries, Parks, Water and Environment (DPIPWE) under scientific permits FA11050, FA11208, FA11295, FA12048 and FA13060.

### Study sites

**Fig 1:**
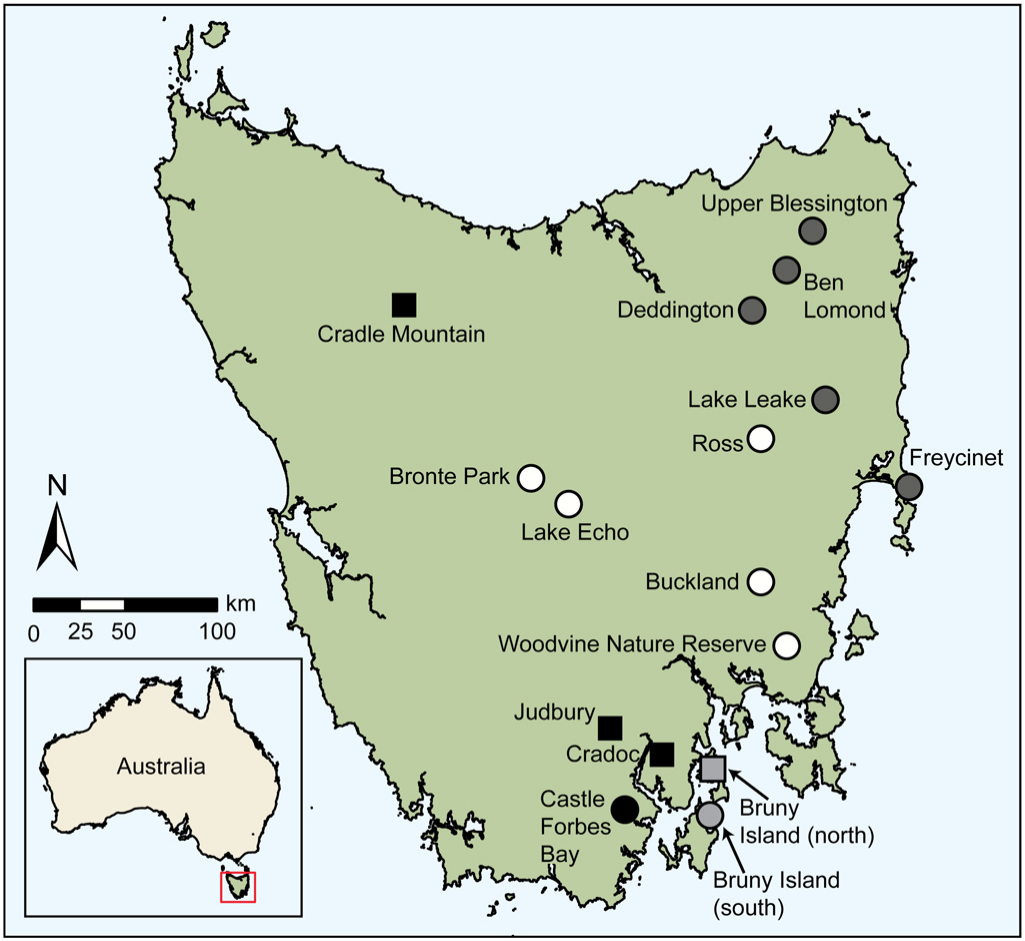
Location of study sites in Tasmania. Circles indicate sites used for statewide camera surveys, squares indicate sites used for longitudinal trapping and camera surveys. Shading indicates DFTD arrival time in region as defined in Hollings, et al. [27] (dark grey—early DFTD arrival (1996–1999); white—mid DFTD arrival (2000–2003); black—late DFTD arrival (2004–2007); pale grey—devil free island). Site location coordinates are listed in Tables 1 and 2. Inset shows location of Tasmania within Australia.

We performed longitudinal trapping and remote camera surveys at four Tasmanian study sites (‘trapping sites’): Cradoc (CR), Judbury (JU), Cradle Mountain (CM) and North Bruny Island (BI) (Fig. 1, Table 1). We categorised each site as ‘declined’ (CR, JU and CM) or ‘stable’ (BI) based on the population status of eastern quolls at the site. The population status for three sites (CR, CM, BI) was determined during a pilot study undertaken in 2010 [35, 40]. The JU site was initially categorised as ‘stable’ based on consistent sightings from longitudinal spotlight surveys [41] and captures from initial trapping surveys during 2011 (this study), but was reclassified to ‘declined’ in early 2012 following unexpected rapid population decline. CR and JU sites were private cattle grazing properties comprising large cleared areas interspersed with intact dry sclerophyll forest. The BI site was located within a large private sheep grazing property that comprised open areas of improved pasture interspersed with remnant dry sclerophyll forest. The CM site was located in the Cradle Mountain-Lake St. Clair National Park and comprised a mosaic of cool temperate rainforest, wet eucalypt forest, mixed forest, buttongrass (*Gymnoschoerus sphaerocephalus*) moorlands and native grasslands.

**Table 1:** Longitudinal population monitoring sites: locations, classifications used for data analyses and key environmental data.

Site	Site code	Location	DFTD region ^a^	Devils present/ absent ^b^	Quoll population status ^c^	Altitude (m asl)	Mean annual precipitation (mm)
Cradle Mountain	CM	41°38′35″S, 145°57′32″E	L	P	Declined	820–950	2360
Cradoc	CR	43°06′13″S, 147°02′40″E	L	P	Declined	80–140	740
Judbury	JU	43°01′24″S, 146°54′50″E	L	P	Declined	255–275	840
North Bruny Island	BI	43°09′48″S, 147°21′17″E	X	A	Stable	30–70	670

^a^ DFTD regions as per Hollings, et al. [27]: E—early disease arrival (1996–1999); M—mid disease arrival (2000–2003); L—late disease arrival (2004–2007); X—devil-free island.

^b^ Devils: P—present; A—absent

^c^ Quoll population status: CM, CR and BI sites categorised as ‘declined’ or ‘stable’, based on pilot study undertaken in 2010 [35, 40]. JU site initially categorised as ‘stable’ based on consistent longitudinal spotlight surveys [41] and initial trapping surveys in 2011 (this study), but reclassified to ‘declined’ in early 2012 following rapid population decline.

**Table 2:** Statewide camera survey sites: locations, classifications used for data analyses and key environmental data.

Site	Site code	Location	DFTD region ^a^	Devils present/ absent ^b^	Quoll abundance high/low ^c^	Altitude (m asl)	Mean annual precipitation (mm)
Ben Lomond	B	41°29′26″S, 147°33′16″E	E	P	High	540–640	850
Bronte Park	BP	42°04′26″S, 146°28′16″E	M	P	High	715–820	950
Buckland	BL	42°31′32″S, 147°39′03″E	M	P	Low	310–365	640
Castle Forbes Bay	CFB	43°07′23″S, 146°56′30″E	L	P	High	205–330	880
Deddington	DE	41°33′43″S, 147°26′38″E	E	P	Low	295–340	750
Freycinet	FR	42°07′35″S, 148°18′38″E	E	A	Low	10–60	690
Lake Echo	LE	42°09′38″S, 146°40′22″E	M	P	Low	865–905	810
Lake Leake	LL	41°53′25″S, 147°46′57″E	E	P	High	650–690	550
Ross	RO	42°02′05″S, 147°34′46″E	M	P	Low	250–300	490
South Bruny Island	SBI	43°18′28″S, 147°18′57″E	X	A	Low	5–30	1090
Upper Blessington	UB	41°28′38″S, 147°35′44″E	E	P	High	435–500	920
Woodvine Nature Reserve	WNR	42°47′14″S, 147°42′48″E	M	P	Low	200–250	660

^a^ DFTD regions as per [Hollings, et al. [27]]: E—early disease arrival (1996–1999); M—mid disease arrival (2000–2003); L—late disease arrival (2004–2007); X—devil-free island.

^b^ Devils: P—present; A—absent

^c^ Quoll abundance: sites categorised as high or low abundance based on statistical differences in Royle Nichols abundance estimates (S1 Table)

We also conducted remote camera surveys at 12 additional sites across the eastern half of Tasmania (‘statewide sites’) (Fig. 1, Table 2) within the eastern quoll’s core distribution which includes Bruny Island (Fig. 1). Eastern quolls are predominantly associated with interfaces between forest habitat used for denning and open grasslands used for foraging [42]. Accordingly, each survey site comprised a structural interface between forest (dry or wet eucalypt forest, mixed forest, eucalypt plantation or tall coastal scrub) and adjacent open areas (pasture or native grasslands, buttongrass plains, harvested or immature (<1 m height) plantation or low open coastal shrub and heathland complexes). As eastern quolls are found in a diverse range of vegetation types [35, 43–45], we considered vegetation structure more important than vegetation type in the current study.

### Trapping surveys

We surveyed eastern quolls and Tasmanian devils at each trapping site using live capture and release. Any feral cats captured were removed and euthanased upon first capture. CR and JU were surveyed every second month from May 2011 to July 2012, with further surveys in January, May and July 2013. CM was surveyed every second month from May 2011 to September 2013 (except November 2012). BI was surveyed every second month from May 2011 to November 2013. We captured animals using standard PVC pipe traps baited with raw lamb heart. Traps were set within a 15 ha study area at CR, JU and BI, with traps strategically placed along the interface between the forest and adjacent open pasture. At CM, traps were set within a 200 ha study area, with traps positioned along the interface between forest and adjacent buttongrass plains or adjacent to trees or shrubs along roadsides within the open buttongrass areas. Survey effort at CR, JU and CM was 90 trap nights per survey. At BI, survey effort was usually 90 trap nights, however due to high capture rates during peak times of year, trap effort was reduced in some surveys to minimise the time quolls were kept in traps prior to processing. We marked each captured quoll or devil with an Allflex ISO-compliant FDX-B passive integrated transponder, recorded the animal’s sex and age, and released the animal at the point of capture.

### Camera surveys

We performed a three-week remote camera survey at each of the 12 statewide sites between mid-July and early November 2012. To eliminate seasonal differences between sites, we performed surveys at the time of year when quoll populations are most stable, thereby avoiding intra-annual fluctuations in eastern quoll populations that occur during the mating season (May-June) and juvenile emergence (late November-February) [46]. The order in which sites were surveyed was designed to ensure similar sunrise and sunset times among regions, thereby ensuring region was not confounded with daylight length. For each survey, we deployed 20 RECONYX PC-800 passive infrared motion-detector cameras for a minimum of 21 nights. Of the three carnivore species, the eastern quoll has the smallest home range of between 35 and 44 ha [42]. To investigate species interactions at the scale occupied by eastern quolls, we positioned cameras ca. 100 m apart along a linear 2 km transect that followed a structural interface between open grasslands and forest. Each camera was fastened to a tree ca. 1.5 m above the ground, with a muttonbird (*Puffinus tenuirostris*) oil scent lure positioned 2–3 m in front of the camera. The camera was aimed at the ground beneath the lure, and additional muttonbird oil was drizzled on the ground in the centre of the frame. For each movement trigger, we programmed cameras to take three pictures in rapid succession, with images taken in further groups of three until movement ceased. An infrared flash was used to illuminate images at night. All images were stamped with the time, date, site and camera number. All observations of carnivore species were recorded for each survey. To minimise repeat captures of the same individual, we only treated a single detection event or ‘activity’ as independent if it occurred >10 minutes after the last series of images for that species on that camera, unless individuals were distinguishable by unique pelage patterns or colours.

To corroborate trapping observations, we also conducted camera surveys at the four trapping sites. Each site was surveyed on three occasions: February/March 2012, June/July 2012 and December 2012/January 2013. Additional surveys were conducted at JU in October 2012, April/May, June and October 2013, and at CM in April, July and September 2013. For each survey, we set 20 cameras for a minimum of 21 nights using the same protocol adopted for the statewide camera surveys. However, given the key aim of these surveys, camera placement at these sites followed the transect lines used in the trapping surveys. Accordingly, these camera surveys were not directly comparable to the statewide surveys.

### Data analysis

All statistical analyses were performed in R version 3.0.1 [47].

#### Number of carnivores trapped

We compared the mean number of individual eastern quolls trapped per survey among sites using a one-factor analysis of variance (ANOVA). For this analysis, we included all survey periods from May 2011 to July 2013 but excluded data from months where surveys were not performed at all four sites during that month. Significant differences between individual sites were identified using a Tukey’s pairwise comparison. We then compared the number of quolls trapped over an annual cycle to identify any seasonal effect. For this analysis, we pooled data from the three declined quoll sites and compared the mean number of quolls trapped per survey to data from the stable quoll site for all bimonthly surveys between July 2011 and July 2012 using a two-factor repeated measures ANOVA.

We compared the mean number of devils trapped among sites using a one-factor ANOVA, and a Tukey’s pairwise comparison was performed to identify which sites differed. As feral cats were only captured at the JU site and were removed when captured, we excluded cats from this analysis.

#### Relative abundance of carnivores

We used the camera survey data from the 12 statewide survey sites to estimate the relative abundance of eastern quolls, feral cats and Tasmanian devils at each site. For each species, we created site-specific detection histories by recording presence or absence for each camera night. We defined a camera night as the 24-hour period from 12:00:00 (midday) to 11:59:59 am on the following day. As cameras at each site were not spatially independent, we pooled detections across all 20 cameras and defined a species as ‘present’ on a given camera night if it was detected on at least one of the 20 cameras at that site that night. We used an occupancy modelling approach [48] to account for the possibility that a species was present but not detected, based on the species-specific detection history for each site. To estimate relative abundance of each species, we used the Royle Nichols (RN) model [49] in the unmarked package version 0.10–3 [50]. The RN model is an extension of the MacKenzie, et al. [48] occupancy modelling approach, which recognises that variation in a species’ abundance induces variation in that species’ detection probability, and exploits this variation to estimate the relative abundance of the species at each site [49]. For this analysis, we incorporated lure age (the number of days since the camera lure was deployed) as a covariate on detection probability.

We used ordinary least squares regression to determine the mean numerical relationship between devil and cat abundance across the 12 statewide camera sites. To examine whether there was any evidence of devils imposing a limiting effect on cat abundance, we used the quantreg package version 5.05 [51] to examine the relationship between devil and cat abundance at the 50^th^, 75^th^, 95^th^ and 99^th^ quantiles using quantile regression. The same approach was used to investigate whether there was any evidence that cat and quoll abundance was negatively related or whether cats limit the upper abundance of quolls.

To investigate the potential for emerging trophic cascades with declining devil abundance, we also compared the abundance of devils, cats and quolls among DFTD regions. We categorised each of the statewide camera sites into early, mid or late DFTD arrival regions based on the year the disease was first reported in the region, using the same categories as Hollings, et al. [27] (Table 2). As Bruny Island is a devil-free island, we excluded the South Bruny Island (SBI) site from this analysis. We then compared the mean abundance of each species among DFTD regions using a one-factor ANOVA.

We also compared sites with high quoll abundance to sites with low quoll abundance to investigate if cat abundance was higher at sites with low quoll abundance. Sites were categorised as ‘high quoll’ or ‘low quoll’ sites based on significant differences in RN abundance estimates. Multiple pairwise comparisons were performed between sites using the unmarked package, with significance levels adjusted using the Bonferroni correction (*α/n*) to reduce the likelihood of type I error. As the Bonferroni correction could be considered too conservative for some analyses [52], we corrected for alpha-inflation using *n* = 11 (for 11 comparisons between 12 sites) rather than *n* = 66 (for all 66 possible pairwise comparisons). Using this adjustment, sites separated into two distinct groups such that abundance at every ‘high quoll’ site was significantly higher than every ‘low quoll’ site. The ‘high quoll’ or ‘low quoll’ categorisation is listed for each site in Table 2. The relative abundance of cats and quolls was then compared between ‘high quoll’ and ‘low quoll’ sites using a one-factor ANOVA.

#### Spatial activity

To investigate the potential for spatial separation among carnivore species, we investigated whether cats were absent from sites where devils were present, and whether quolls were absent from sites where cats were present.

#### Temporal activity

To investigate the potential for temporal separation among carnivore species, we used the timestamp recorded on remote camera images to create temporal activity profiles for each species, using the overlap package version 0.2.3 [53]. We fitted non-parametric kernel density curves using default smoothing parameters to characterise the probability density distribution of each species’ activity pattern. The smoothing parameter (1/*c*) is the inverse of the concentration parameter (*c*) of the von Mises kernel (corresponding to a circular distribution) for a given sample; increasing the smoothing parameter above 1.0 produces a flatter kernel density curve while reducing it below 1.0 provides a more ‘spiky’ curve [54]. For small sample sizes, Ridout and Linkie [33] found that a default parameter of 0.8 minimises any over or undersmoothing of the data, thereby minimising any effect on the resulting estimators of overlap. For each species or site category pair, we then calculated the coefficient of overlapping, Δ [55], as a measure of total overlap between the two species’ estimated distributions. This measure ranges from 0 (no overlap) to 1 (complete overlap) and is defined as the area under the curve that is formed by taking the minimum of the two density functions at each time point. Due to the low number of cat detections in some analyses, we used the Δ_1_ measure recommended for small sample sizes [33] and obtained 95% confidence intervals from 10,000 smoothed bootstrap samples after accounting for bootstrap bias [54].

For each species or site category pair, we also used the non-parametric Mardia-Watson-Wheeler test in the circular package version 0.4–7 [56] to test for homogeneity in species activity profiles. This test detects differences in the mean angle of the circular temporal data indicative of differences in activity peaks, and requires a minimum of 10 detections for each species [57]. This test assumes no repeat data, so records with identical timestamps were altered by 0.001 degrees (0.24 seconds) in the raw data.

To investigate the potential for devils to affect the temporal distribution of cat activity, we analysed activity profiles for the 11 statewide camera survey sites by DFTD region (excluding the devil-free SBI site). We also compared activity profiles of cats between sites where devils were present (*n* = 10) and those where devils were absent or undetected (*n* = 2) and also between early and mid DFTD regions. To investigate the potential for cats to temporally suppress quoll activity, we compared activity profiles of quolls and cats at high quoll sites (*n* = 5) with those at low quoll sites (*n* = 7). To examine whether this potential changed seasonally, we compared activity profiles between cats and quolls in February, June and December 2012 at the CR site. The number of cat detections at JU, CM and BI were too low to perform a similar seasonal comparison at these sites.

## Results

### Number of carnivores trapped

We trapped significantly more individual eastern quolls per trapping survey at the stable quoll site (mean ± standard error: 30.00 ± 3.56) than at the declined quoll sites (4.85 ± 0.57) (*F*_1,2_ = 5.62 x 10^2^, *P* = 0.002). The number of quolls trapped at the declined sites did not differ significantly among sites (all *P* > 0.758).

**Fig 2:**
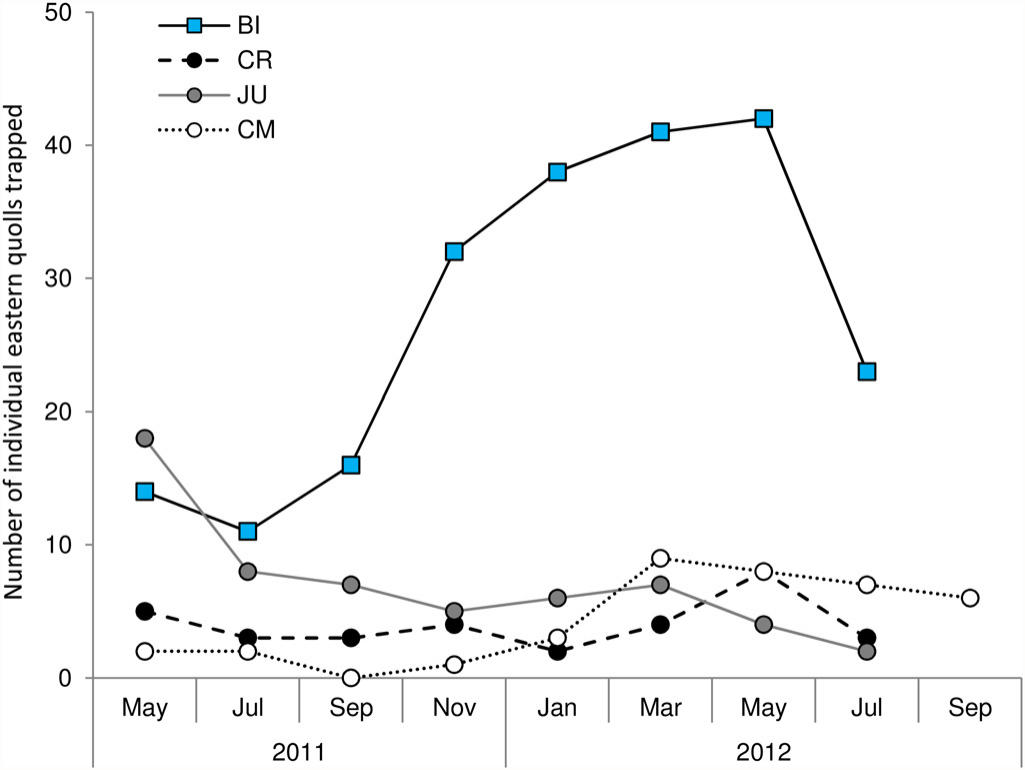
Number of individual eastern quolls captured at longitudinal trapping survey sites. Trap effort for all sites was 90 trap nights per session, except BI November 2011 (55 trap nights). North Bruny Island (BI, blue squares); Cradoc (CR, black circles); Judbury (JU, grey circles); Cradle Mountain (CM, white circles).

Across the 2011–2012 annual cycle, we found a significant interaction between survey month and quoll population status (*F*_5,14_ = 9.66, *P* < 0.001), with a distinct seasonal effect evident at the stable quoll site, but not at the declined quoll sites (Fig. 2). The number of quolls trapped at the stable site in July and September increased markedly in November, and remained high until May, before decreasing again in July. We did not find any evidence of a similar marked increase at the declining sites in November, where quoll captures remained low throughout the year.

**Fig 3:**
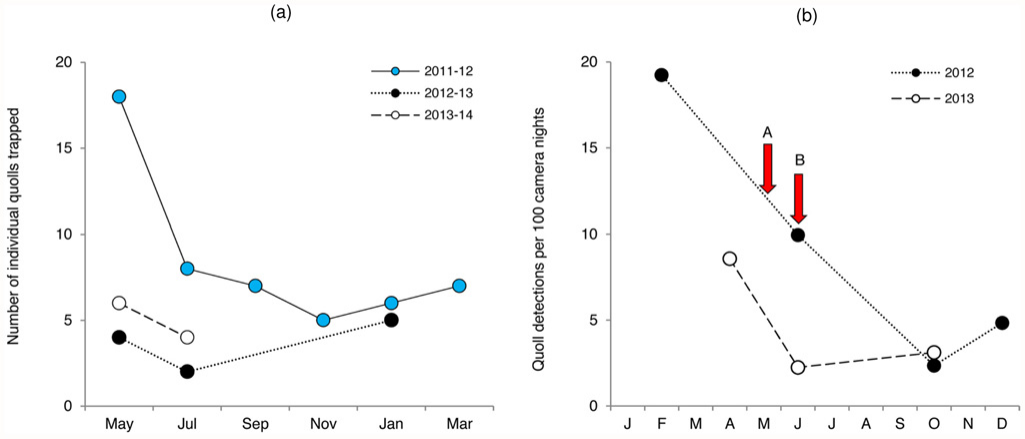
Reduction in the number of eastern quoll detections at Judbury. Plots show (a) number of individual quolls trapped per trapping survey; and (b) number of quoll detections per 100 camera nights in camera surveys. Survey effort comprised (a) 90 trap nights per survey; and (b) 20 cameras set for a minimum 21 nights. Arrows indicate the point when feral cats were first detected in trapping surveys (A) and in camera surveys (B). Trapping surveys (a) for 2011–12 were performed prior to first feral cat detection at the site; 2012–13 and 2013–14 surveys were performed after feral cats were first detected.

The number of quolls trapped at JU declined markedly between 2011–12 and 2012–13 (78% decline from May 2011 to May 2012; 63% decline from July 2011 to July 2012) and remained low thereafter (Figs. 2 and 3a). Similar declines in quoll detections were observed over the seven camera surveys conducted at this site between February 2012 and October 2013 (Fig. 3b). Cats were first trapped and removed from the site in May 2012 (*n* = 3). There were further captures and removals in July 2012 (*n* = 1), May 2013 (*n* = 1) and July 2013 (*n* = 1). Cats were first detected on camera in June 2012 (Fig. 3b) and, despite their ongoing removal, additional detections were made in October 2012, May, June and October 2013. The number of devils captured at JU did not differ between years.

Both trapping and camera surveys detected devils at all trapping sites except BI. As expected, significantly more devils were trapped at JU than at BI where devils are absent (*P* = 0.018), however the number of devils trapped did not differ between other sites (all *P* > 0.074). Cats were not trapped at any site except JU, although they were detected on camera at all four trapping sites.

### Relative abundance of carnivores

**Fig 4:**
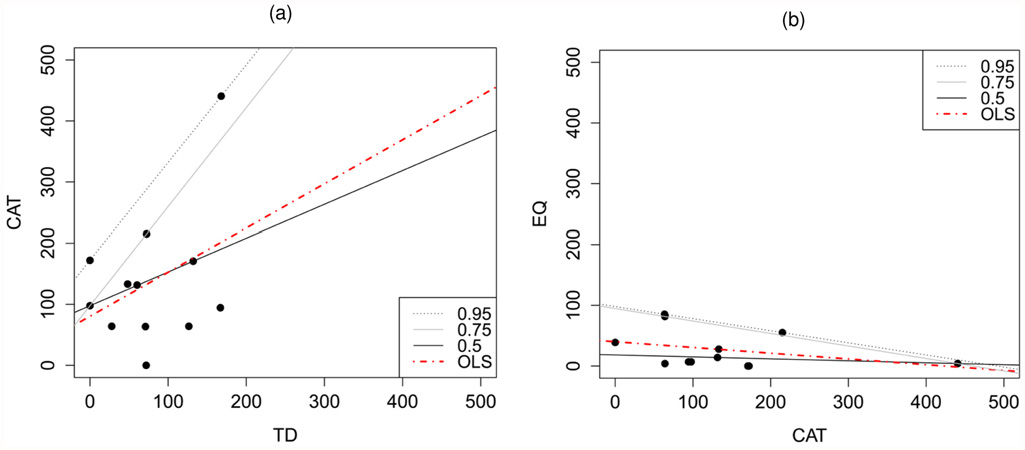
Relationship between estimated abundance of predators at statewide camera survey sites. Plots show abundance of (a) Tasmanian devils (TD) and feral cats (CAT); and (b) feral cats and eastern quolls (EQ). Each data point represents Royle Nichols abundance estimates for each species for a single camera survey site (*n* = 12 sites) as listed in Table 2. Regression lines shown for 50^th^ quantile (0.5—black, solid), 75^th^ quantile (0.75—grey, solid), 95^th^ quantile (0.95—black, dotted) and ordinary least squares (OLS—red, dot-dashed). For both figures, the lines for the 95^th^ and 99^th^ quantiles were identical, so only the 95^th^ quantile line is shown.

**Fig 5:**
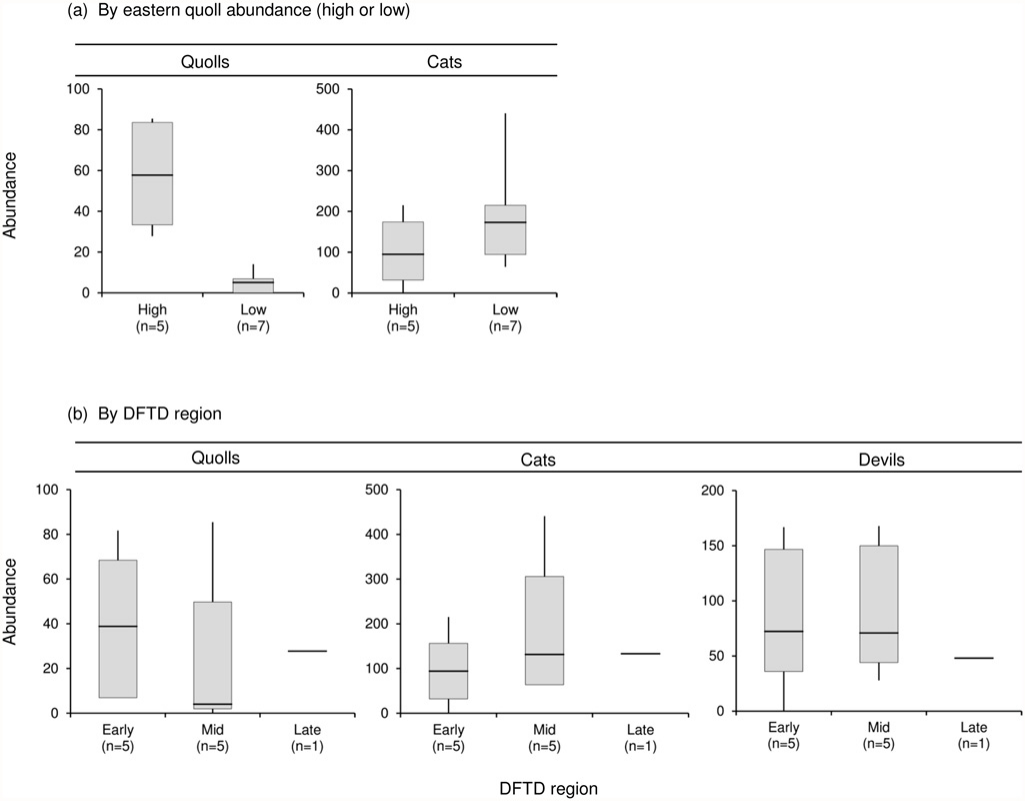
Mean abundance estimates for eastern quolls, feral cats and Tasmanian devils from statewide camera survey sites. Sites grouped by (a) high/low quoll abundance (*n* = 12 sites); and (b) DFTD arrival region (*n* = 11 sites). Sites categorised into high/low quoll abundance and DFTD regions as per Table 2. Analysis by DFTD region at (b) excludes data from SBI (devil-free island). Box boundaries enclose the 25^th^ and 75^th^ percentiles, horizontal bar is the mean, whiskers indicate maximum and minimum values. Sample sizes in parentheses indicate number of sites.

Among the statewide camera survey sites, observed cat abundance was not negatively related to devil abundance (*F*_1,10_ = 1.62, *P* = 0.231) and we did not find any evidence that devils limited the upper limit of cat abundance at any of the assessed quantiles (all *P* ≥ 0.145; Fig. 4a). Similarly, quoll abundance was not associated with cat abundance among the statewide camera sites (*F*_1,10_ = 1.30, *P* = 0.282) and we did not find any evidence of cats limiting the upper abundance of quolls at any of the assessed quantiles (all *P* ≥ 0.385; Fig. 4b). We found that while quoll abundance differed significantly between high and low quoll sites (*F*_1,10_ = 29.5, *P* < 0.001), there was no difference in cat abundance (*F*_1,10_ = 1.23, *P* = 0.294) (Fig. 5a). Abundance estimates and 95% confidence intervals are listed for all species for all sites in S1 Table.

We did not find any evidence of trophic cascades in abundance following devil declines, with no difference in the relative abundance of quolls (*F*_2,8_ = 0.29, *P* = 0.757), cats (*F*_2,8_ = 0.52, *P* = 0.611) or devils (*F*_2,8_ = 0.22, *P* = 0.805) among DFTD regions (Fig. 5b).

### Spatial activity

We did not find any evidence that the presence of devils had a negative effect on local cat presence. Cats were detected at 92% (12 of 13) of camera or trapping sites where devils were detected, indicating that both species were locally active in these areas. Similarly, we did not find any evidence for local spatial separation of quolls and cats. Quolls were detected at 87% (13 of 15) of camera or trapping sites where cats were recorded.

### Temporal activity

**Fig 6:**
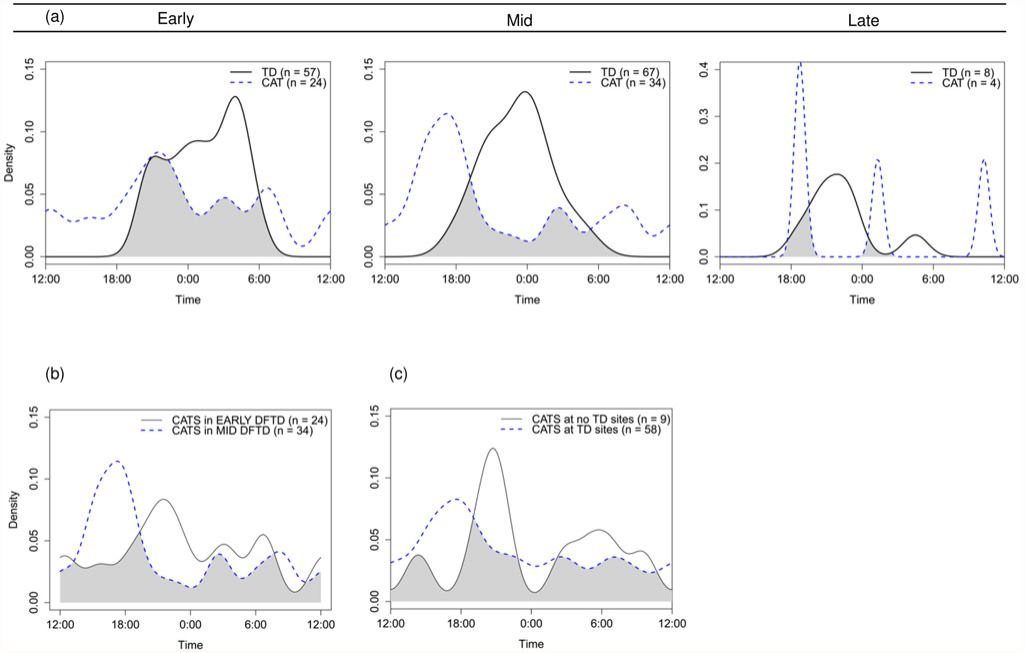
Activity of devils and cats from statewide camera survey sites. Plots at (a) show overlap of devil (TD, black solid line) and cat (CAT, blue dashed line) activity, grouped by DFTD arrival region. Sites (*n* = 11) categorised into DFTD regions as per Table 2 (excludes data from SBI (devil-free island)). Care should be taken in interpreting results from the late DFTD region due to the low number of detections. Plot at (b) shows difference in cat activity between early (black solid line) and mid (blue dashed line) DFTD regions (*n* = 10); and (c) shows difference in cat activity between sites with devils present (blue dashed line) and sites with devils absent (black solid line). For (c), sites (*n* = 12) categorised into devils present or absent as per Table 2. Sample sizes in parentheses indicate number of detection events for each species. Grey shading indicates the overlap in species activity.

We found evidence of temporal separation between cats and devils (Fig. 6a). Cat activity in the late DFTD region demonstrated marked separation from devil activity (Δ_1_ = 0.18), although as only one site (20 cameras) was located in this region, the low number of detections precluded the calculation of confidence intervals and the performance of the Mardia-Watson-Wheeler test. Accordingly, care should be taken in further interpreting results from this region. Reduced separation was evident in the mid DFTD region (Δ_1_ = 0.42 (95% CI: 0.24–0.51)), with distinct separation between peaks in cat activity (around sunset) and devil activity (peaking around midnight) (*W* = 43.84, *P* < 0.001). Separation was less evident in the early DFTD region where devils had declined the longest; total overlap in activity was higher (Δ_1_ = 0.60 (0.43–0.75)), and both cat and devil activity peaked nocturnally, although peaks occurred at different times of night (*W* = 11.11, *P* = 0.004).

Differences in cat activity between early and mid DFTD regions (Δ_1_ = 0.63 (0.44–0.80); *W* = 7.75, *P* = 0.021; Fig. 6b) were similar to differences in cat activity observed between sites with and without devils (Δ_1_ = 0.62 (0.41–0.85); Fig. 6c). Cat activity peaked around sunset in the mid DFTD region and at sites where devils were present, but peaked nocturnally in the early DFTD region and at sites where devils were absent. As there were less than 10 cat detections at sites where devils were absent, we were unable to perform the Mardia-Watson-Wheeler test for the comparison between sites with and without devils.

**Fig 7:**
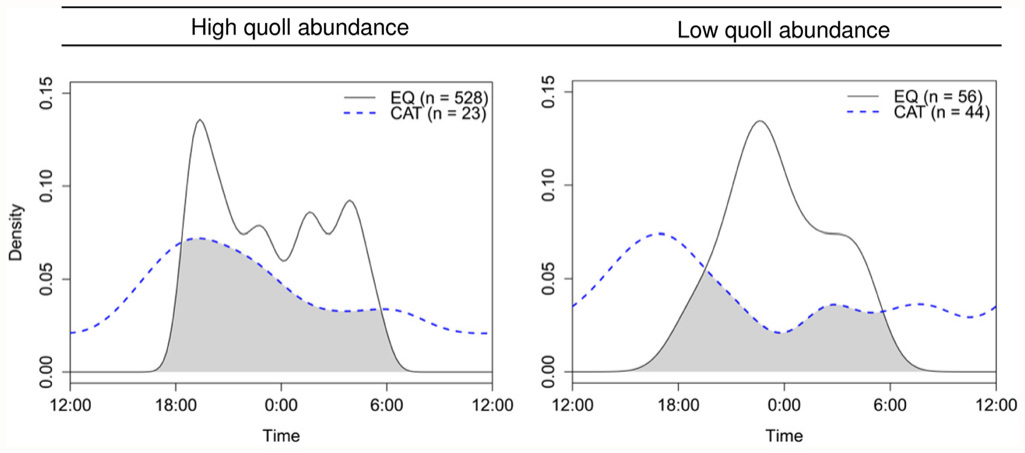
Overlap of eastern quoll and feral cat daily activity from statewide camera survey sites. Sites categorised as high (*n* = 5 sites) or low (*n* = 7) quoll abundance as per Table 2. Plots show overlap of quoll (EQ, black solid line) and cat (CAT, blue dashed line) activity. Sample sizes in parentheses indicate number of detection events for each species. Grey shading indicates the amount of temporal activity overlap between quolls and cats.

Quoll activity was strictly nocturnal at all statewide camera sites, however the temporal activity profiles differed between high and low quoll sites (Fig. 7). At high quoll sites, activity peaked following sunset, and quolls remained fairly active until sunrise. At low quoll sites, the peak following sunset was notably absent, and activity peaked around midnight. Cats were active across both day and night, with a similar activity peak around sunset at both high and low quoll sites (Fig. 7). Differences in cat and quoll activity were evident at both high (*W* = 6.42, *P* = 0.040) and low quoll sites (*W* = 40.20, *P* < 0.001). There was increased total overlap between cat and quoll activity at high quoll sites (Δ_1_ = 0.62 (95% CI: 0.46–0.76)) compared to low quoll sites (Δ_1_ = 0.48 (95% CI: 0.31–0.57)).

**Fig 8:**
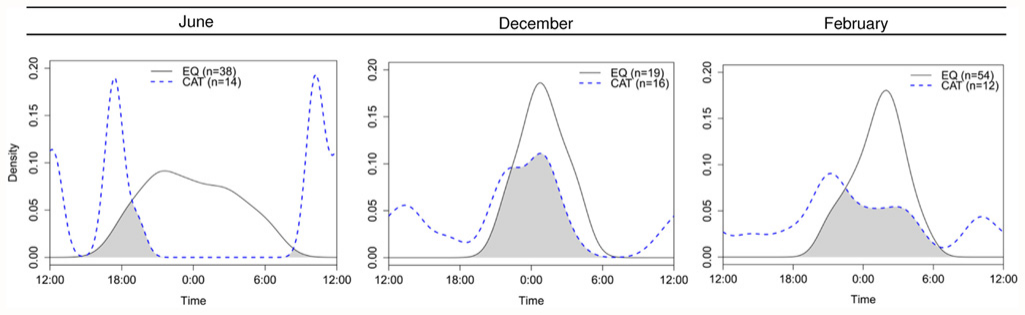
Seasonal overlap of eastern quoll and feral cat daily activity at Cradoc in 2012. Plots show overlap of quoll (EQ, black solid line) and cat (CAT, blue dashed line) activity. Sample sizes in parentheses indicate number of detection events for each species. Grey shading indicates the amount of overlap in temporal activity between quolls and cats.

At CR, cat and quoll activity differed in February (*W* = 10.32, *P* = 0.006) and June (*W* = 27.56, *P* < 0.001) but not in December (*W* = 2.29, *P* = 0.319) (Fig. 8). The overlap between cat and quoll activity differed seasonally (Fig. 8). In winter, cat activity was largely crepuscular and diurnal, exhibiting minimal overlap with nocturnally active quolls (Δ_1_ = 0.21 (95% CI: 0.08–0.28)). In summer, cat activity was predominantly nocturnal, resulting in increased overlap with quoll activity in both December (Δ_1_ = 0.58 (95% CI: 0.37–0.80)) and February (Δ_1_ = 0.51 (95% CI: 0.28–0.73)).

## Discussion

Our findings suggest that devils influence feral cat behaviour, but contrary to our prediction, we did not find any evidence that devils suppress cat abundance (Fig. 4a) and there was no evidence of increased cat abundance in areas where devils had declined the longest (Fig. 5b). As we predicted, observed cat and devil activity separated temporally, with separation less evident in areas where devils had declined the longest (Fig. 6a). Cat activity was more nocturnal in areas where devils had declined the longest (Fig. 6b). This apparent shift presents an emerging threat to nocturnal competitors and potential prey species that may have infrequently encountered cats prior to DFTD.

Contrary to our predictions, we did not find evidence to support a negative relationship between cat and quoll abundance (Fig. 4b). The overlap in cat and quoll activity was greater in areas with higher quoll abundance (Fig. 7). Overlap was also greater over summer than in winter (Fig. 8), implying a high risk of predation for juvenile quolls. We suggest that while cats do not appear to have caused the recent quoll decline, predation of juvenile quolls by cats could be inhibiting low density quoll populations from recovering their former abundance through a ‘predator pit’ effect [58, 59]. Predation intensity could increase further should cats become increasingly nocturnal in response to devil declines.

### Devil and cat interactions

Devil and cat abundance did not differ among DFTD regions (Fig. 5b) and we did not find any evidence that devils suppress the abundance of cats (Fig. 4a). Devil abundance did vary among sites within each DFTD region (S1 Table), but the similarity in mean devil abundance among regions could indicate that, below a certain density, DFTD transmission rates are reduced. This accords with findings of the Save the Tasmanian Devil Program (Sam Fox, STTDP, pers. comm.): relatively consistent, very low devil numbers with reduced disease prevalence, have been trapped in areas where DFTD has long been present. At the time of our surveys, DFTD had been present in the study region for between 5 and 16 years.

The similarity in cat abundance among regions was unexpected. There are two likely explanations. First, if devils were suppressing cat abundance prior to DFTD, the high reproductive capacity of feral cats [60] might have allowed rapid increase in cat abundance following the decline of devils, so that current abundance could reflect the ‘post-release’ abundance across regions, and the similarity in cat abundance could reflect the similarity in devil abundance among regions. If this is the case, cat abundance appears to have plateaued at new equilibrium levels across DFTD regions, with no apparent effect of time since devil decline at our survey sites (Fig. 5b). While we did not find any evidence for devils suppressing or limiting cat abundance (Fig. 4a), it is possible that devil densities could now be too low to be affecting cats across our survey sites, although Saunders [28] did not find evidence of suppression at DFTD-free sites supporting high devil densities in north-west Tasmania. However, in the absence of reliable cat abundance data prior to DFTD arrival in these regions, we are unable to ascertain if current cat abundance differs from pre-DFTD abundance. An alternative explanation is that devils do not suppress cat abundance, but rather other factors, possibly bottom-up processes, could be more important in determining cat abundance, as shown by Hollings, et al. [27] for some regions. Different conditions promote or inhibit the transmission of predatory effects, including predator diversity, strength of interactions, ecosystem productivity, presence of refuges and the potential for compensation [1, 61–64]. For example, top-down processes might be more pronounced where there are strong productivity gradients such as in the high arctic or in arid environments, where food is limiting and competition for scarce resources is high [62, 65], while predator removal in highly productive environments can result in weak effects that do not cascade through trophic levels [66]. Accordingly, Tasmania’s overall higher productivity [67] might promote only weak competitive interactions between devils and cats, thereby dampening any potential mesopredator release following decline of devils. Weak competitive interactions have been observed between large predators and mesopredators in other systems, such as coyotes (*Canis latrans*) and racoons (*Procyon lotor*), although the conditions necessary for these species’ coexistence are not understood [68]. Furthermore, the prey size range and feeding ecology of devils and cats is also quite different, with devils (carnivore/scavengers) [22] unlikely to reduce or limit the availability of smaller live prey species typically hunted by opportunistic predatory cats [69, 70].

The temporal partitioning of observed cat and devil activity suggests that cats could be avoiding devils. With the exception of the early DFTD region, cats were typically crepuscular or diurnal and their activity was largely separated from the nocturnally active devils (Fig. 6a). In the early DFTD region where devil populations had declined the longest, cats were more nocturnal, exhibiting an increased overlap with devil activity (Fig. 6a). In the absence of temporal activity data for cats and devils prior to DFTD arrival in these regions, we are unable to determine if regional differences in temporal activity are a response to declining devils, or if these differences already existed prior to DFTD arrival. However, the differences in observed cat activity between the early and mid DFTD regions (Fig. 6b) are similar to the differences in observed cat activity at sites where devils were present compared with sites where devils were absent (Fig. 6c). This supports the suggestion that observed differences between regions could be a response to declining devils. Further studies are needed in disease-free areas to investigate activity profiles of devils and cats prior to DFTD arrival, and to monitor if and how carnivore activity changes as DFTD spreads through the region.

The apparent response of cat activity to reduced devil abundance involves a delay, which we did not predict. A delayed response by cats could reflect the persistence of innate anti-predator responses to devils, even after selective pressures have been relaxed. For example, black-tailed deer (*Odocoilus hemionus sitkensis*) retained innate anti-predator responses to wolves (*Canis lupus*) during a ca. 100 year period of predator absence [71]. Such behaviours could persist in the absence of a predator due to the low fitness costs associated with the behaviour [72]. Given the high availability of alternative abundant prey sources in Tasmania, avoidance of nocturnally active devils is unlikely to result in reduced fitness for cats. However, selective triggers, such as the drought endured in Tasmania during the three years to 2008 [73, 74], could have been sufficient to increase that cost due to reduced food availability, and therefore might have forced cats to extend their hunting activities nocturnally in an effort to find limited food resources. With reduced devil abundance and reduced interference competition, nocturnal activity would now impose minimal costs to cats, enabling them (and subsequently their kittens) to specialise on nocturnal prey [75], resulting in the gradual shift in cat activity over a few generations. Even in the absence of increasing cat abundance, temporal shifts in cat activity would present an increased predation risk for nocturnally active species such as eastern quolls that may have rarely encountered cats prior to devil decline.

Higher spotlight sightings of cats identified by Hollings, et al. [27] in the early DFTD region could reflect an increase in detectability rather than an increase in abundance. We did not find any evidence of higher abundance (Fig. 5b), but the increased nocturnal activity of cats observed in the early DFTD region (Figs. 6a and 6b) would likely make the cats more detectable during spotlighting surveys, which take place at night. Furthermore, while we did not find evidence of cats avoiding devils spatially in the current study, our statewide camera surveys were not performed along roads where spatial avoidance might be more evident. If devils suppress cat behaviour through interference competition, cats may have historically avoided roads where devils forage for road kills [76], resulting in devils being detected, but cats less likely to be detected in vehicle-based spotlight surveys conducted along roads [77]. Following devil decline, cats might now be more active along roads and therefore more detectable in road-based spotlight surveys [77]. Indeed, Lazenby and Dickman [31] found that devils can alter the detectability of cats along vehicular trails and roads, with the probability of detecting a cat often more than double at sites where devils were not detected than at sites where devils were detected. Future studies analysing GPS-movement data from sympatric devils and cats are needed to better understand the spatial interactions between these species at finer spatio-temporal scales than can be assessed using either camera or spotlight surveys.

The differing interpretations between Hollings, et al. [27] and this study will, in part, reflect the different collection methods and data analyses adopted. The analysis by Hollings, et al. [27] of statewide spotlighting data was the first study to investigate broader ecosystem effects of devil decline as they relate to a range of trophic levels, using the best available data at that time. However, spotlight surveys are known to be an unreliable method for monitoring abundance of cryptic species such as feral cats [78, 79]. An inherent weakness of spotlight survey data is that a brief snapshot on a single night each year is likely to miss or underestimate activity that will more easily be detected by remote cameras left *in situ* for three continuous weeks. While the use of longitudinal spotlight sightings as an index of abundance does allow comparisons to be made before and after DFTD arrival, such data ignores the importance of detectability [77]. Accordingly, such analyses assume that the non-detection of a species means that the species was absent, whereas a non-detection could simply reflect a behaviour that makes that species less detectable in different places or different times. While longitudinal trends from spotlight surveys have been corroborated with alternative methods such as trapping surveys for devils [23] and eastern quolls [35], a similar comparison has not been performed for cats in Tasmania. Accordingly, it might be premature to presume an increase in cat sightings reflects an increase in cat abundance.

While cats appeared to avoid devils temporally, we did not find any evidence that this apparent shift in activity led to a reduction in cat abundance (Figs. 4 and 6). Mammalian and avian mesopredators that avoid larger predators through temporal separation of activity can suffer reduced fitness consequences from hunting at sub-optimal times of day, with reduced resource availability and increased energy demands often leading to reduced breeding success and survival [6, 80, 81]. Such costs of avoidance could be predicted to translate into reduced abundance over time. However, the similarity in cat abundance between regions with different cat activity profiles suggests that temporal shifts are not detrimental to cat fitness and abundance (Fig. 5). Accordingly, the apparent temporal avoidance strategy adopted by cats might simply reduce their likelihood of antagonistic encounters with devils, as has been suggested with subordinate predators avoiding dominant lions (*Panthera leo*) in Africa’s large predator guild [3], but otherwise provides no net benefit or loss to cat abundance.

### Interactions of cats and eastern quolls

The observed activity profiles of eastern quolls differed between sites with high and low quoll densities, but this was not related to cat activity or abundance (Fig. 7). There was greater temporal overlap between cats and quolls at the high-density quoll sites than at the low density sites, but this was a function of differing quoll activity, with no observed difference in cat activity. Given that the increased overlap was observed at higher quoll density sites, there is no indication that it has resulted in an increased predation risk to quolls. This is further supported by our finding that cat and quoll abundance were not related (Fig. 4b).

The difference in quoll activity between high and low-density quoll sites could reflect differences in intraspecific competition for food. A temporal profile similar to the high density quoll sites was observed in the July 2012 camera survey on BI which supports the only confirmed stable, high density population of eastern quolls in Tasmania. The absence of devils and very low abundance of cats at this island site suggest that quoll activity is unlikely to reflect avoidance strategies in response to perceived threats from larger mammalian predators, although avian predators might still influence quoll activity. Accordingly, the similarity in the profiles between BI and the high quoll density sites on mainland Tasmania suggests that top-down processes are not a primary driver of quoll activity and that bottom-up processes are likely to be important. The delayed peak in activity around midnight at the low density sites likely reflects the reduced quoll activity in response to reduced competition for food at these sites, further supporting this hypothesis. However, to understand the influence of bottom-up processes on quoll activity, further information on the spatial and temporal variation in eastern quoll diet and activity of key prey species would be required.

The consistently low number of quolls trapped and detected at the three declined quoll sites confirms that these populations have shown no sign of recovery (Fig. 2). Further declines were observed in both trapping and camera surveys at the JU site during the course of the study (Fig. 3). This decline in quolls coincided with a rapid and complete decline in detections of the Tasmanian bettong (*Bettongia gaimardi*) at this site, with declines of both species coinciding with the first appearance of cats at the site [82]. A combination of trapping and spotlight surveys failed to detect any cats in bimonthly surveys performed at the site between May 2011 and March 2012 or in a camera survey performed in February 2012. However, once cats were first detected in May 2012, they continued to be frequently detected on camera and regularly trapped (and removed) up to and including the final trapping survey in July 2013 and the final camera survey in October 2013 [82]. It is possible that cats could have been present at the site but undetected prior to May 2012, however this seems unlikely given the consistent results from a range of complementary survey techniques. While the number of quolls detected and trapped dropped rapidly, low numbers of quolls continued to be detected at the site until the end of the study. It might be that quolls at this site were initially naïve to the presence of cats, and were therefore vulnerable to predation when cats first arrived, with surviving quolls learning to avoid cats and enabling a low number of quolls to persist at this site. While these observations suggest that cats could have contributed to both quoll and bettong declines at this site, this evidence is entirely correlative and does not demonstrate causation. The decline in quolls could alternatively reflect bottom-up processes rather than top-down suppression by feral cats. However, as we did not survey prey abundance as part of the current study, we are unable to discern the mechanism(s) responsible for the quoll decline.

While we did not find any association between cats and quolls generally (Fig. 4b), individual cats could have a disproportionate impact. Our statistical assessment assumes that all individuals are ecologically equivalent [83]. Many populations of generalist species, such as feral cats, comprise specialised individuals whose niches are a subset of the population niche [83, 84]. Cats are known to specialise on the type of prey with which they have had prior experience [75] and thus individual cats can exhibit preferences in the types of prey they hunt [85]. For example, Gibson, et al. [86] found that predation by two individual feral cats was catastrophic to vulnerable rufous hare-wallaby (*Lagorchestes hirsutus*) populations released into the Tanami Desert. Once these two individual cats were removed, no further killings occurred during the next 2–3 years, despite the ongoing presence of other cats in the area. Methods such as camera surveys are not appropriate to establish if and how this individual specialisation of cats might influence cat and quoll dynamics, however specialisation by individual cats provides a possible explanation for the observed rapid decline in quolls at JU following cat incursion at this site (Fig. 3). While predation by individual specialist cats remains one candidate agent of local decline, spatial shifts out of the local study area could also have contributed to the observed reduction in quolls at this site. Indeed, two quolls that were frequently captured prior to cat incursion were subsequently recaptured after a 12 month period of no captures following cat arrival. However, as areas surrounding the immediate study site were not monitored in the current study, we are unable to assess the extent to which this might have occurred.

The absence of a summer spike in quoll captures at the three declined sites suggests low or no juvenile recruitment at these sites (Fig. 2). The eastern quoll has a short, highly synchronised mating season each year, resulting in a large influx of newly independent juvenile quolls into the population between November and February each year [46]. Numbers typically start to decline around March and usually reach pre-juvenile abundance by July each year [46]. This characteristic annual cycle was observed at the stable site, but was notably absent at the three declined sites (Fig. 2). Individual female quolls trapped at the declined sites had, on average, more pouch young in July (or September at CM) than quolls at the stable site [39], indicating that individual reproductive output was not reduced. However we are unable to assess if mortality occurred while young were in dens (between August and November) or when they first emerged as independent juveniles. Demographic modelling will be required to assess whether juvenile recruitment is reduced or absent at declined sites, and whether this reflects reduced reproductive success, or mortality of newly independent or emigrating juveniles.

The high summer overlap observed between cat and quoll activity at CR (Fig. 8) does suggest a high risk of predation to juvenile quolls, which could contribute to inadequate recruitment at the declined quoll sites. Cats are known to kill juvenile quolls [87]. For example, two juvenile eastern quolls (360g) were killed (at different locations) from crushing injuries to the thorax and abdomen, with paired canine penetration wounds consistent with attack by a cat (B Fancourt, pers. obs.). The high seasonal overlap of cat and quoll activity observed in December indicates a high predation risk to small (350–600 g), vulnerable juveniles that become independent around that time. A high degree of overlap was still evident in February when immigrating juvenile quolls are most mobile, but had reduced by June when surviving juveniles have reached adult size. The ontogeny of decreasing vulnerability from juveniles in February to adults in May/June is reflected in the anti-predator behavioural response to cats that is exhibited by juvenile but not adult male eastern quolls [88]. There are several reasons why cats might shift their activity seasonally, including prey abundance or activity, environmental temperatures, or avoidance of larger predators. While the drivers of cat activity in this study are not known, such a seasonal shift could present a high risk to juvenile quolls in summer.

A lack of juvenile recruitment at the declined quoll sites could explain why the Tasmanian mainland populations have not recovered. As cats have been in Tasmania for over 200 years [38], it is highly unlikely that cat predation of juvenile quolls presents a new threat to quoll populations. Previously, the formerly high abundance of quolls might have allowed populations to sustain predation of some juveniles without having detrimental impacts on population viability. As quoll populations appear to have recently been driven to very low densities by a period of unsuitable weather, the reproductive capacity of the few persisting individuals at each site may now be insufficient to withstand the same level of predation, with declined populations now trapped in a ‘predator pit’[58, 59]. Small populations are inherently more susceptible to demographic, environmental and genetic stochasticity [89–91]. Our findings at the high density BI site (where there have never been devils) support this hypothesis. While cats were detected during two of the three camera surveys performed at the BI site, quoll densities have remained significantly higher than at all of the declined sites, with the higher reproductive capacity of the large quoll population presumably outnumbering any losses to predation. As we did not find any evidence of cats increasing in abundance with declining devils (Fig. 5b), cat predation of juvenile quolls is also unlikely to have increased following devil decline. However, the apparent delayed temporal shift in cat activity following devil decline (Fig. 6) could further increase cat predation of eastern quolls over time.

### Limitations and future research

We investigated interactions among devils, feral cats and eastern quolls to better understand any potential contribution to the ongoing decline and suppression of eastern quoll populations. Our study builds on the initial work and hypotheses of Hollings, et al. [27] by specifically examining these interactions within the eastern quoll’s distribution across the drier eastern half of Tasmania. The analyses conducted by Hollings, et al. [27] excluded several spotlight regions in core quoll habitat in southern Tasmania and included several spotlight regions in NW Tasmania that fall outside of the core quoll distribution. Therefore, any inferences to be made regarding ecological interactions, in so far as they may be contributing to quoll declines or inhibiting quoll recovery, are limited.

Care should be taken not to over interpret our results from the late DFTD region. As most of the late DFTD region falls outside of the core eastern quoll distribution, only one of our statewide camera sites was located in the region. Our study did not investigate the potential influence of bottom-up processes such as prey activity and abundance, environmental variables and vegetation, but this should be the next logical step. However, as eastern quolls are found in almost all vegetation types excluding large tracts of rainforest [35, 44, 45], the increased survey effort required to achieve the necessary power to detect any differences in low-density populations could be prohibitive.

Our study is the first to investigate potential behavioural interactions among devils, cats and eastern quolls. However, as pre-DFTD data is not available to perform before-after-control-impact (BACI) analyses [92, 93], our ability to infer whether observed differences between DFTD regions are a response to disease-induced devil declines are limited. While such BACI analyses should be performed as the disease moves through regions that are currently DFTD-free, these areas are outside the core distribution of the eastern quoll and hence any new understanding will be limited to interactions between devils and cats.

Future research should also test our hypothesis that eastern quoll populations have been reduced below a sustainable threshold from which they are unable to recover without management intervention. Even in the absence of any increase in threat following the decline in devils, the inherent nature of small populations and their potentially ineffective population size means that natural recruitment might not be high enough to overcome established levels of threat. It may be necessary to establish insurance populations of eastern quolls, to repopulate local areas where eastern quolls have declined, with populations monitored to assess their ability to persist in the face of current, ongoing threats.

## Supporting Information

S1 Table Estimated abundance of Tasmanian devils, feral cats and eastern quolls across 12 statewide camera sites. Estimates calculated using Royle Nichols model [49].
(DOCX)
