# Supplementary material for: Devil Declines and Catastrophic Cascades: Is Mesopredator Release of Feral Cats Inhibiting Recovery of the Eastern Quoll?
Source: PLoS One. 2015 Mar 11;10(3):e0119303. doi: 10.1371/journal.pone.0119303 (PMC4356622; doi:10.1371/journal.pone.0119303)
Supplement: S1 Table — Estimates calculated using Royle Nichols model [49]. (DOCX) [file pone.0119303.s001.docx]

|  | Tasmanian devils | | Feral cats | | Eastern quolls | |
| --- | --- | --- | --- | --- | --- | --- |
| Site | Estimated abundance | 95% confidence intervals | Estimated abundance | 95% confidence intervals | Estimated abundance | 95% confidence intervals |
| B | 72 | 57-88 | 0 | 0-0 | 39 | 28-50 |
| BL | 168 | 144-192 | 441 | 401-481 | 4 | 2-7 |
| BP | 71 | 56-87 | 63 | 49-79 | 85 | 69-99 |
| CFB | 48 | 36-62 | 133 | 111-156 | 28 | 20-37 |
| DE | 167 | 143-191 | 94 | 76-114 | 7 | 4-11 |
| FR | 0 | 0-0 | 98 | 79-117 | 7 | 4-11 |
| LE | 28 | 19-38 | 64 | 49-80 | 4 | 2-7 |
| LL | 126 | 106-148 | 64 | 49-80 | 82 | 66-97 |
| RO | 132 | 111-154 | 171 | 146-196 | 0 | 0-0 |
| SBI | 0 | 0-0 | 172 | 147-198 | 0 | 0-0 |
| UB | 72 | 57-89 | 215 | 187-244 | 55 | 42-69 |
| WNR | 60 | 46-75 | 132 | 110-154 | 14 | 9-20 |
